# New-type urbanization and regional public health: mechanisms and effects

**DOI:** 10.3389/fpubh.2025.1513173

**Published:** 2025-04-24

**Authors:** Jinfang Wang, Xin Luo, Xian Liang, Caiwang Ning

**Affiliations:** ^1^Institute of New Rural Development, Jiangxi Agricultural University, Changbei Economic and Technological Development Zone, Nanchang, China; ^2^School of Economics and Management, Beijing Forestry University, Beijing, China; ^3^Jiangxi Regional Development Research Institute, Jiangxi University of Technology, Nanchang, China

**Keywords:** new-type urbanization, economic agglomeration, population agglomeration, regional public health, spatial spillovers

## Abstract

New-type urbanization (NTU) in China not only effectively promotes socio-economic transformation but also serves as a significant driving force for the coordinated development of regional social and environmental demands. Using data from China spanning 2007–2018, this study examines the effects and mechanisms of NTU on regional public health (RPH) by constructing panel fixed-effects, threshold-effects, moderated-effects, and spatial spillover models. The findings are as follows: ① NTU plays a significant role in promoting RPH. Threshold analysis reveals distinct threshold effects for employment density and industrial structure sophistication. ② Socio-economic agglomeration plays an important moderating role in the relationship between NTU and RPH. Specifically, there is a significant substitution effect between economic agglomeration and NTU in affecting RPH, while population agglomeration improves NTU’s positive effect. ③ Across the eastern, central, and western regions, NTU has a positive impact on RPH, with the strength of influence increasing progressively. However, in economically developed regions, the effect is non-significant. ④ NTU shows significant spatial spillover effects on RPH, with indirect effects exceeding direct effects. The main factor influencing NTU’s inhibitory effect on RPH is whether regions are adjacent.

## 1 Introduction

With the acceleration of globalization and modernization, urbanization has become a common developing trend worldwide, while new-type urbanization (NTU) is expected to be a driving force for China’s next round of economic transformation. It involves not only the migration of the population from rural to urban areas but also the optimization of the urban spatial structure, the transformation and upgrading of the economic structure, and the diversification of social and cultural development. In this context, socio-economic development is closely interconnected with urbanization, and the impact on regional public health (RPH) is also becoming more and more prominent and has become a hot issue of social concern. Rapid global urbanization poses challenges to sustainable development (1), and new-type urbanization is a key pathway for sustainable social development (2). A typical feature of urbanization is the concentration of population (3), and its impact on economic transformation can either stimulate or hinder the socio-economic development of the region. In the process of NTU, the expansion of the urban scale, the increase in population density, and the lifestyle change have had a profound impact on public health services, environmental quality, and the health behavior of residents. Environmental degradation and population overload caused by rapid urban growth seriously threaten human and regional health (4). The agglomeration effect^1^ of urbanization promotes the concentration of healthcare resources and the improvement of healthcare services. At the same time, urbanization problems such as environmental pollution, traffic congestion, and work pressure exacerbate the risk of chronic and infectious diseases and other public health risks.

Additionally, RPH is not only related to the overall wellbeing and living quality of the region’s residents but is also key to promoting economic development and social stability and reducing health inequalities. How NTU affects RPH is relevant to socio-economic development and the enhancement of human wellbeing and is of great significance in achieving sustainable regional development. Therefore, exploring the mechanism of NTU’s impact on RPH is important for formulating scientific and rational urban planning and public health policies.

Previous research has mainly focused on the measurement of NTU levels and their impact on the economy, society, and environment. Measurement of NTU provides a basis for related research, with the majority of available studies deriving from its intrinsic definition, where indicators consist of single and multidimensional dimensions (5). The single dimension follows the characteristics of traditional urbanization, which is characterized by population urbanization. However, compared with the traditional population urbanization featuring a household registration system, NTU pays more attention to social welfare, ecological wellbeing, and social culture. Within the multidimensional measurement indicators, factors such as economic development, social welfare, infrastructure development, and the urban–rural income gap are included (6). Population, economic, and social urbanization are also commonly included in these multidimensional indicators (7). Some studies further incorporate spatial urbanization into the index system, constructing an NTU measurement framework encompassing four dimensions: population, economic, social, and spatial.

Additionally, urban–rural integration and environmental urbanization have gradually attracted the attention of researchers and have been included in the measurement system. Research in this field primarily focuses on the provincial level (8, 9) and the municipal level (10–12). It has been shown that NTU affects not only population mobility and economic and social development but also the ecology and the environment (8, 13). Notably, urbanization is a significant factor contributing to changes in the urban environment and the emergence of human health problems (14).

The rise of NTU as an important part of socio-economic planning has attracted extensive attention from researchers. Rapid urbanization has important implications for public health and may continue to increase the population’s exposure to major risk factors for disease (15). Meanwhile, the improvement of healthcare facilities and services, environmental protection awareness, and economic development that accompany urbanization are also positively affecting public health. One of the important transmission pathways is energy use. Energy is considered a source of economic growth, which can effectively improve living quality and health; however, it is accompanied by a series of environmental problems that threaten public health (16).

Fossil fuel consumption is an important contributor to climate change and environmental pollution, and NTU can influence energy intensity and efficiency in the region (6, 17). Further, it affects the emission of pollutants, harmful and greenhouse gases in the process of industrialization (8, 18) and effectively ameliorates haze pollution (19, 20). The enhancement of energy-saving and environmental protection technologies, economic growth, industrial structure upgrading, and technological advancement are also positive impacts of NTU (6). Moreover, NTU promotes high-quality urban development by enhancing green total factor productivity, green-intensive land use, and industrial agglomeration (10, 11).

Although a large number of studies have explored the relationship between NTU and RPH, the following research gaps still exist: First, the existing research has mostly explored the two-by-two relationship between NTU, socio-economic development, and RPH, but rarely has it incorporated all three into the same analytical framework. Logically, NTU is related to multiple economic, social, demographic, and environmental aspects, which are important factors that may affect health and wellbeing in the area. Secondly, existing studies tend to spotlight the direct impact of NTU on local public health while ignoring its possible spatial effects. Namely, NTU affects local public health and may also have an impact on the public health situation in neighboring or economically well-connected regions through channels such as economic linkages and population mobility. Third, different regions facing different challenges and opportunities in NTU also show significant heterogeneity in their public health. However, existing studies often adopt a one-size-fits-all approach and fail to fully consider the differences and specificities between regions, making it difficult for the results to accurately reflect the true impact of new urbanization on RPH.

This study is intended to bridge the above research gaps and delve into the mechanisms by which NTU affects RPH, with particular attention to spatial spillover effects and regional heterogeneity. Specifically, this research will first extend the analytical framework of public health-influencing factors from the perspective of NTU and test the effect of NTU on RPH and the moderating role of socio-economic agglomeration. Second, spatial econometric methods are used to quantify the impact of NTU on the public health of adjacent or economically connected regions, revealing their spatial interdependence and spillover effects. Third, based on the economic development level, demographic structure, industrial structure, and other characteristics of different regions, the heterogeneity test is used to explore the differences in the impacts of NTU on RPH and to provide a scientific basis for the formulation of differentiated public health policies.

This study is intended to deepen the understanding of the relationship between NTU and RPH, to provide policymakers with more comprehensive and precise decision support, and to promote the coordinated development of NTU and public health.

Possible marginal contributions: first, it extends the analytical framework of public health-influencing factors from the perspective of NTU. Second, NTU, socio-economic development, and RPH should be integrated into one analytical framework to verify NTU’s public health effects. Third, the effects and scale of NTU on RPH and the moderating effect of socio-economic agglomeration are empirically tested. Finally, the spatial effects of NTU on RPH are verified from the perspective of spatial spillovers.

## 2 Theoretical mechanism and hypothesis proposal

### 2.1 Direct mechanism and threshold effect of new-type urbanization on public health

According to the theory of health production, health status depends on the combined effect of various health input factors. Health is a commodity jointly produced by a series of input factors such as lifestyle, living environment, education, income level, and medical services. In the process of new-type urbanization, all of these factors will change significantly, thus affecting the health status of residents. Specifically, NTU focuses on comprehensive human development, emphasizes urban–rural integration, industry-city integration, ecological livability, and sustainable development, and focuses on the transformation of the economic development model, optimization of the social structure, and enhancement of ecological environmental protection (21, 30). Not only does the transformation bring new vitality to socio-economic development, but it also results in a more profound implication for RPH. NTU has a multifaceted influence on RPH, leading to both positive changes and accompanying challenges (15).

First, NTU promotes the optimal allocation and centralized utilization of healthcare resources. NTU promotes a high concentration of resources, capital, and professionals in specific regions (19). However, there may be a threshold effect^2^ on the economic development level, meaning that NTU cannot exert an upgrading effect on individuals and RPH when the economic level is lower. Following the economic growth, the government and society’s investment in public health has been increasing. The allocation of medical and health resources is further optimized, and medical service quality is continuously improved, resulting in the improvement of RPH (22).

Second, from the development concept of NTU, it is committed to building a more livable urban environment (22, 23). In NTU processing, the government has increased the investment in urban infrastructure, public service facilities, and the environment to improve the quality of urban residents. In particular, in terms of industrial development, the green transformation of industry is being promoted through continuous encouragement of industrial structure upgrading. Industrialization is considered a cause of environmental pollution, as NTU leads to industrial agglomeration and the generation of large quantities of pollutants, further weakening the health effects. Therefore, there may be a threshold effect of industrial structure in NTU affecting RPH. With the upgrading of the industrial structure, NTU gradually strengthens the investment in public health services and continuously improves the living conditions of residents, which further promotes the enhancement of RPH.

Third, in terms of resource allocation, NTU’s construction has promoted urban–rural integrated development, broken the urban–rural dichotomy, and promoted the allocation of public health resources (22, 33). By constructing a series of rural medical and health service systems, the government has made it possible for rural residents to benefit from the same public health services as urban residents, narrowing health disparities in both urban and rural areas.

Summarizing the above, NTU provides a boost to RPH through the combined effects of public health infrastructure construction, optimization of the environment, and promotion of urban–rural integration. Accordingly, the following hypotheses are proposed:

*H1:* New-type urbanization can significantly promote regional public health.

*H1a:* There is a threshold effect of socio-economic development level in the promotion of regional public health by NTU.

*H1b:* There is a threshold effect of advanced industrial structure in the promotion of regional public health by NTU.

### 2.2 Moderating effect of socio-economic agglomeration

Socio-economic agglomeration, as a concentration within socio-economic behaviors, reflects the vitality and potential of the economy, which is an important factor affecting public health (5, 30). In new-type urbanization, the economic effect is significantly enhanced with the concentration of population capital production factors (5, 19). Socio-economic agglomeration can amplify the positive impacts of urbanization on public health. The agglomeration effect helps to optimize resource allocation and improve production efficiency, thus providing a more solid economic foundation for the supply of public health services (21, 24). When regional economic agglomeration increases, the government and the community may invest more sufficiently in public health. The optimization of health resources and improvement of service quality becomes possible, which contributes to the improvement of RPH. The mitigation of the negative impacts of urbanization on public health. Problems such as environmental pollution and stressful living conditions that urbanization may bring about can be mitigated in economic agglomeration areas through technological means and policy interventions. In addition, economic agglomeration accelerates knowledge and technology innovation and may also bring about air pollution control effects (34, 35).

Economic agglomerations have a high concentration of enterprises, research institutes, and similar actors, creating an environment conducive to knowledge spillover and technological innovation. It also helps to improve residents’ health literacy and disease prevention awareness. Meanwhile, economic agglomeration also promotes technological innovation and service change in healthcare (36), providing residents with more convenient and efficient healthcare services and promoting the RPH. In addition, it can indirectly promote RPH by improving urban infrastructure and the environment. With the development of growing economic agglomeration, the urban infrastructure during NTU is improving, while the ecological environment is effectively protected, providing a more livable living environment for residents (37). Improving this environment helps minimize the health hazards of environmental pollution for the population, decreases the incidence of disease, and improves their quality of life.

In summary, economic agglomeration enhances the promotion of RPH by NTU through promoting efficient resource allocation, accelerating knowledge and technology innovation, and improving urban infrastructure and the ecological environment. On this basis, the following hypothesis is proposed:

*H2a:* Economic agglomeration plays a positive moderating role from NTU to RPH.

Population agglomeration significantly affects carbon emissions and PM2.5 (38–40), as well as constantly influencing the urban–rural development gap (41). When NTU affects RPH, population urbanization effectively improves the health literacy and self-care ability of the overall population in NTU by facilitating the dissemination of information and knowledge sharing. It will help reduce the incidence of diseases and improve RPH. As the population gathers, the demand for healthcare services in NTU increases accordingly, which prompts increased investment in healthcare to enhance healthcare coverage. The innovation of healthcare service is further promoted, and new service models such as community healthcare and telemedicine are developed to meet the health needs of residents (19, 30). During NTU, the effect brought by population agglomeration promotes the upgrading and renovation of urban infrastructure, including public health facilities, fitness, and leisure facilities, which provides residents with more convenient and comprehensive health services (14). Improving residents’ quality, helping prevent and control diseases, and maintaining RPH.

In summary, population agglomeration enhances NTU’s contribution to RPH by accelerating the dissemination of information, optimizing the allocation and efficiency of healthcare resources, and promoting infrastructure for public health. Accordingly, the following hypothesis is proposed:

*H2b:* Population agglomeration plays a positive moderating role from NTU to RPH.

### 2.3 Spatial spillover effect

**Figure 1:**
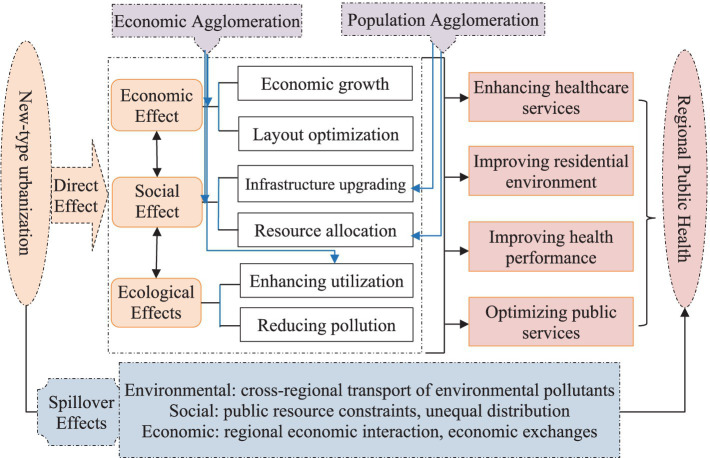
Framework diagram.

In the process of NTU, the agglomeration of resources, capital, manpower, and others led to a relative lack of resources in adjacent regions and an uneven distribution of medical and healthcare resources. When the NTU advances rapidly, it attracts and gathers a large number of high-quality medical resources, while adjacent regions may face an inadequate supply of medical services due to the outflow of resources, which may affect public health. In addition, following the expansion of cities and the acceleration of industrialization, environmental pollution has become a prominent problem. Cross-regional transmission of air, water, and other pollutants leads to a decline in the environmental quality of neighboring regions, thus increasing the risk of respiratory and cardiovascular diseases among residents. As a result, the environmental pollution problems brought about by NTU have negative spillover effects on neighboring regions (Figure 1).

Furthermore, NTU will also have a negative impact on RPH in adjacent regions indirectly through population movement. As NTU advances, some rural residents migrate to urban areas, but they may not change their living habits and health awareness, and such unhealthy lifestyles are spread to residents in adjacent areas. Moreover, neighboring regions are subjected to environmental quality degradation caused by trans-regional pollution transmission, which negatively affects public health in adjacent regions. Accordingly, the following hypothesis is proposed:

*H3:* NTU has negative spatial spillovers on public health in adjacent regions.

## 3 Research design

### 3.1 Modeling

#### 3.1.1 Benchmark regression

Fixed-effects models can effectively control for individual heterogeneity (such as geographic location and cultural practices) that does not vary over time when studying panel data, thereby allowing for a more accurate estimation of the effect of the independent variable on the dependent variable. In this study, considering the inherent differences between regions that may impact the relationship between NTU and RPH, employing a fixed-effects model can eliminate the effects of these non-time-varying factors, resulting in more robust estimates.

Based on the theoretical analysis in the previous section, in order to empirically test the relationship, the following fixed-effects model controlling for individual and time effects is constructed:


(1) $$PH_{\mathrm{it}}=\beta_{0}+\beta_{1}NTU_{\mathrm{it}}+\beta_{2}Con_{\mathrm{it}}+\mu_{i}+\vartheta_{t}+\varepsilon_{\mathrm{it}}\text{,}$$

where *i* is the region; *t* denotes the year; $PH_{\mathrm{it}}$ is the public health level of the region; NTU represents the degree of NTU; Con represents the set of control variables; β is the coefficient to be estimated. To further minimize the impact that omitted variables may have on the robustness of the results, $\mu_{i}$ and $\vartheta_{t}$ are added to the model as province-fixed and time-fixed, respectively. $\varepsilon_{\mathrm{it}}$ is a random perturbation. Same below.

#### 3.1.2 Threshold effect model

Considering that NTU may have a non-linear effect on RPH through labor force employment density and the degree of industrial structure advancement, a panel threshold model is constructed based on Hansen’s (1999) threshold test model, as follows:


(2) $$\begin{array}{l} PH_{\mathrm{it}}=\alpha_{0}+\alpha_{1}NTU_{\mathrm{it}}\times I\left(M_{\mathrm{it}}\leq \tau_{1}\right)+\alpha_{2}NTU_{\mathrm{it}}\\ \times I\left(\tau_{1}<M_{\mathrm{it}}\leq \tau_{2}\right)+\alpha_{3}NTU_{\mathrm{it}}\times I\left(\tau_{2}<M_{\mathrm{it}}\right)\\ +\alpha_{g}Con_{\mathrm{it}}+\mu_{i}+\vartheta_{t}+\varepsilon_{\mathrm{it}}\text{,} \end{array}$$

In Equation 2, $M_{\mathrm{it}}$ is the threshold variable in period t; $I\left(�\right)$ represents the schematic function; $\tau_{i}$ is the threshold parameter value. Other characterizations are the same as in Equation 1.

#### 3.1.3 Spatial estimation model

To further analyze the correlation between RPH and neighboring regions, the spatial correlation in each region was explored by using Moran’s *I*, and the model is set up as follows:


(3) $$\mathrm{Mora}n^{’}s\;I=\frac{n\overset{n}{\underset{i=1}{\sum }}\overset{n}{\underset{j=1}{\sum }}w_{i,j}z_{i}z_{j}}{S_{0}\overset{n}{\underset{i=1}{\sum }}{z_{i}}^{2}}$$

$$S_{0}=\overset{n}{\underset{i=1}{\sum }}\overset{n}{\underset{j=1}{\sum }}w_{i,j},z_{i}=x_{i}-\bar{x}\text{,}$$

In Equation 3, $w_{i,j}$ is spatial weight matrix; $S_{0}$ is the aggregation of all spatial weights, and $z_{i}$ denotes the deviation of an individual from the mean.

NTU not only affects local public health but may also affect the public health of neighboring or economically connected regions through spatial spillover effects. Spatial spillover models capture this spatial interdependence and spillover effects and are essential for understanding the full impact of NTU on RPH.

Drawing on the Likelihood Ratio Test, Wald and Lagrange Multiplier Test, and Hausman, the study constructs SPDM. The modeling setup is shown as follows:


(4) $$\begin{array}{l} PH_{\mathrm{it}}=\beta_{0}+\beta_{1}NTU_{\mathrm{it}}+\beta_{2}Con_{\mathrm{it}}+\lambda_{1}WNTU_{\mathrm{it}}\\ +\lambda_{2}WCon_{\mathrm{it}}+\rho WPH_{\mathrm{it}}+\mu_{i}+\vartheta_{t}+\varepsilon_{\mathrm{it}}\text{,} \end{array}$$

In Equation 4, $WNTU_{\mathrm{it}}$ and $WPH_{\mathrm{it}}$ are the spatial lag terms of NTU and RPH, respectively; λ represents estimated spatial regression coefficients.

In this study, fixed-effect models are used to control for individual heterogeneity and to ensure that estimates of the impact of NTU on RPH are not influenced by factors that do not vary over time, thereby improving the accuracy of the estimates. The spatial model, meanwhile, is used to capture the possible spatial spillover effects of NTU and reveal its potential impact on public health in adjacent or economically connected regions. The combined use of these two models controls for the effects of non-time-varying factors and accommodates spatial interdependence, providing a comprehensive perspective for a deeper understanding of the relationship between NTU and RPH.

### 3.2 Variables

#### 3.2.1 Dependent variable

Regional public health (RPH) was chosen as the explanatory variable in this study. The RPH index was used to characterize RPH. Regarding its calculation, the entropy method was employed to measure RPH. Public health encompasses both physical and mental health; however, due to data availability, this study focuses on seeking proxy variables to represent public health through the lens of physical health. The public infrastructure security system is a prerequisite for public health and medical facilities, personnel, and expenditures were selected for characterization. At the macroscopic level, the status of public health is visualized by the population’s survival rate and mortality rate. Furthermore, large-scale outbreaks of public health issues are commonly manifested as infectious disease outbreaks, and the incidence rate of infectious diseases is more indicative of public health status compared to other negative indicators. The variables are defined in Table 1.

**Table 1:** Variables definitions.

Type	Indicators	Definition	Direction
Public health	Basic Support Conditions	Number of beds in medical and health institutions per 10,000 people	+
		Number of doctors per 10,000 people	+
		Per capita financial expenditure on healthcare	+
	Health Performance Improvement	Provincial survival rate	+
		Provincial Population Mortality Rate	−
		Incidence rate of legally reported infectious diseases of categories A and B	−
New-type urbanization	Population urbanization	Urban population density (people/km^2^)	+
		Urbanization rate of resident population (%)	+
		Urban registered unemployment rate (%)	−
	Economic urbanization	Share of secondary industry in GDP (%)	+
		Share of tertiary industry in GDP (%)	+
		Per capita disposable income of urban residents (yuan)	+
	Social urbanization	Participation rate of urban residents in basic pension insurance (%)	+
		Higher education students per 100,000 people	+
		Road area per capita (m^2^)	+
		Number of public transportation vehicles per 100,000 people (standard units)	+
	Urban–rural integration	Ratio of per capita income in urban and rural areas	−
		Ratio of per capita consumption expenditure of urban and rural residents	−
		Engel’s coefficient of urban and rural residents	−

#### 3.2.2 Independent variable

The independent variable in our studies is new-type urbanization (*NTU*). Generally, it aligns with the concepts of comprehensive sustainable development outlined in *China’s National New-type Urbanization Plan (2021–2035)*. Based on previous research (25, 26), the index system includes four primary indexes and 13 s-level indexes. The variables are defined in Table 1, and the degree of NTU is measured using TOPSIS.

#### 3.2.3 Moderating variables

Socio-economic agglomeration was selected as a moderating variable, which includes economic agglomeration and population agglomeration, respectively. Socio-economic development is often accompanied by economic and population mobility, where economic and population agglomeration is a typical form of socio-economic agglomeration and may affect public health. Referring to existing research (27), this study measures the degree of population agglomeration by comparing the ratio of regional population density (population/area) to national population density (population/area of the country). Economic agglomeration generally implies the aggregation of economic activities; therefore, economic density is selected in this study to characterize the degree of economic agglomeration. This means assessing the degree of the sparseness of economic activities in spatial extent, measured as the ratio of the added value of non-agricultural industries in the region to the area of the region.

#### 3.2.4 Control variables

By combining research on RPH, this study identified other possible influences. Relevant variables were controlled to improve the accuracy of the results. Control variables were drawn from socio-economic development, energy consumption, scientific and technological development, and infrastructure development. Specifically, GDP per capita is defined as the logarithm of the city’s real GDP divided by the regional population, while energy consumption is expressed as standard coal used per 10,000 yuan of GDP. Technological innovation is represented by S&T expenditures as a share of fiscal expenditures, and road infrastructure development is measured using the ratio of year-end real road area to the administrative district.

### 3.3 Data description

(1) Reliability and representativeness of data sources and variables. In this study, the raw data for the variables and indicators are primarily sourced from the China Health Statistics Yearbook, China Statistical Yearbook, China Environmental Statistics Yearbook, China Energy Statistics Yearbook, and the statistical bulletins from various regions for the corresponding years, published by different statistical departments of the government and reputable research institutes. The reliability of these data sources is comparatively high. The selection of variables considers their definitions, the realistic context, and existing studies while also accounting for the reliability of data collection in practice before finalization. Overall, the data sources and variables are both scientific and reliable.
(2) Reliable data quality control measures. First, verify the data sources to ensure that all data originate from authoritative and reliable databases. For unofficial or third-party data, confirm their accuracy and consistency by cross-verifying them through multiple channels. Regarding some missing data, during the collection process, comparisons were made with government work reports and statistical bulletins in each region to ensure the sources were reliable. Subsequently, standardized data collection methods and processes are used to maintain data consistency and comparability. Data processing is conducted in strict accordance with predetermined cleaning, organizing, and conversion rules to prevent data distortion or misuse. Finally, the data are validated to check for logical errors, missing data, or outliers. Outliers are eliminated, corrected, or processed using appropriate statistical methods based on the specific context to ensure the authenticity and validity of the data.
(3) The chosen time range (2007–2018) and regions for the study are appropriate. First, regarding data availability and completeness, the relevant statistics for 2007–2018 are comprehensive and easily accessible. Next, this period marks an important phase in China’s urbanization development, and NTU has significantly influenced public health, making it an ideal timeframe to examine the relationship between NTU and public health. Finally, urbanization policies at the national level remained relatively stable during this period, which helped eliminate the interference of policy changes in the study results. In addition, the impact of the global COVID-19 pandemic on public health was also taken into account, as it may amplify or diminish the effect of NTU on public health. In summary, selecting 2007–2018 can maximize the validity and accuracy of the results.

Regarding the suitability of regional selection, 30 provinces in China (excluding Tibet) are representative, covering various regions across eastern, central, and western China. This selection helps reveal both regional differences and common characteristics in the impact of NTU on public health. Additionally, all provinces in NTU face similar public health challenges, such as environmental pollution, traffic congestion, and insufficient public health provision. By studying the data from each province, a more comprehensive understanding of the overall impact of NTU on public health can be achieved. Finally, the study’s results can provide valuable references for policymakers nationwide to better address the public health issues that arise during NTU. Moreover, the findings can also provide guidance for the practical operations of each province to promote the coordinated development of urbanization and public health.

In summary, the data for the indicators involved in this study are primarily sourced from the respective years of the *China Health Statistics Yearbook*, the *China Statistical Yearbook*, the *China Environmental Statistics Yearbook, the China Energy Statistics Yearbook,* and statistical bulletins from various regions. Any missing values have been filled in using interpolation. Moreover, considering data availability, 30 provinces (including cities and districts) in China from 2007 to 2018 were finally selected as the research sample. As shown in Table 2, the study sample is deemed appropriate through the construction of an econometric model.

**Table 2:** Descriptive statistical.

Variable	Symbol	Obs	Mean	Std. Dev.	Min	Max
Regional public health	*RPH*	360	0.425	0.103	0.205	0.788
New-type urbanization	*NTU*	360	0.392	0.139	0.064	0.853
Energy use	*EU*	360	0.939	0.516	0.24	3.461
S&T input	*TP*	360	1.974	1.383	0.389	7.202
Government regulation	*GR*	360	23.108	9.794	8.704	62.686
Road infrastructure construction	*RIC*	360	0.231	0.338	0.001	1.749
Openness to the outside	*OP*	360	1.429	17.201	0.012	313.964

## 4 Results and discussions

### 4.1 Benchmark regression

#### 4.1.1 Results of the benchmark regression

Based on the analysis above and the Hausman test, this study employs a fixed-effects model to examine how NTU affects RPH. The consistency of the results obtained by including and excluding the control variables is tested separately, with Columns (1) and (2) (Table 3) presenting the estimation results without and with the variables, respectively. The estimation results indicate that the regression coefficient of NTU on public health is positive, suggesting that the development of NTU significantly enhances RPH, thus supporting Hypothesis 1. Furthermore, the regression coefficient value shows that for every 1% increase in urbanization, RPH improves by 0.193%. The control variables of energy consumption intensity, technological innovation, and road infrastructure construction also contribute to the RPH level to some extent, indicating that the inclusion of these variables is warranted (Table 4).

**Table 3:** Results of the benchmark regression.

Variables	*RPH*		Variables	*RPH*	
	(1)	(2)		(1)	(2)
*NTU*	0.722***	0.193***	*RIC*		0.231***
	(22.48)	(4.08)			(7.14)
*EU*		−0.068***	*OP*		−0.000
		(−4.98)			(−0.19)
*S&T Input*		0.016***	*Constant*	0.142	0.175***
		(3.28)	
(11.13)		(2.77)
*GR*		0.007***	*R^2^*		
	(8.35)		
0.606	0.791

*t*-statistics in parentheses, ***p < 0.01. Same below.

**Table 4:** Robustness test results.

Variables	(1)	(2)	(3)	(4)
	Replace the core explanatory variable	Excluding municipalities	2007–2014	OLS
*NTU*	0.720***	0.400***	0.145***	0.494***
	(8.54)	(6.83)	(2.75)	(13.55)
*Constant*	−0.120**	0.160***	0.203***	0.008
	(−2.05)	(3.97)	(4.23)	(0.18)
*Controls*	Yes	Yes	Yes	Yes
*Province FE*	Yes	Yes	Yes	Yes
*Year FE*	Yes	Yes	Yes	Yes
*Observations*	360	312	240	360
*R^2^*	0.820	0.804	0.679	0.706

***, **, and * represent significance levels of 1%, 5%, and 10%, respectively.

The impact of NTU on public health has two aspects. First, urbanization leads to population and economic agglomeration, which may create a “crowding effect” on RPH resources, thereby reducing RPH. Additionally, urbanization intensifies industrialization, producing a significant amount of industrial exhaust that exacerbates air pollution and creates serious environmental issues, which can severely threaten the health of the population and further threatens human health (41). It has been demonstrated that NTU significantly enhances employment quality for agricultural migrants (42) and reduces carbon emission intensity. In addition, NTU increases the agglomeration effect, improves resource utilization efficiency, and enhances public health service facilities.

The “agglomeration effect” of urbanization surpasses the “crowding effect, “indicating that the socio-economic development and welfare brought by urbanization have improved RPH. In promoting NTU, attention must be paid to balancing development and environmental protection in order to achieve sustainability.

#### 4.1.2 Robustness testing

The above regression indicates that NTU can significantly contribute to RPH, and the following approaches are selected for robustness testing: firstly, replacing explanatory variables. In the research, the metrics of NTU encompass four aspects: economic, social, population, and urban–rural integration. Drawing on existing studies (5), urbanization is measured by population urbanization. Therefore, population urbanization was chosen as the explanatory variable [column (1)]. The estimated coefficients are significantly positive and consistent with benchmark regression. Second, the effect of special areas should be excluded. Because municipalities directly under the central government have special characteristics in China’s administrative regions, this study chooses to repeat the above steps by excluding the four regions of Beijing, Tianjin, Shanghai, and Chongqing. The coefficient of NTU retains the same signature as the benchmark regression, and both are significantly positive [column (2)]. After controlling for special regions, NTU still has a significant driving effect. Thirdly, shortening period. The goal of building NTU was officially proposed in 2014 by China, and policy shocks may affect the health effects of urbanization construction. The data from 2007 to 2014 are selected for robustness testing [column (3)]. The results show that the estimated coefficients are still significantly positive, which is in line with the benchmark regression.

Finally, the estimation model was changed. Instead of relying on the previous method, we used Ordinary Least Squares (OLS) regression [column (4)]. The estimated coefficient of NTU on RPH remains significantly positive, and the regression results after changing the estimation method can be considered robust.

#### 4.1.3 Endogeneity testing

Since NTU, as the core explanatory variable, is not strictly exogenous, this study also faces certain endogeneity challenges. RPH is closely related to the quality of urban development, and public health demand affects the allocation and planning of urban resources, affecting the spatial layout and functional structure of urbanization; thus, the endogeneity problem may be endogenous. The endogeneity problem is addressed by finding effective instrumental variables using the 2SLS model, and the lagged one-period explanatory variables are used as instrumental variables in this study.

Table 5 presents the results of 2SLS estimates based on instrumental variables. Significant correlations in the expected direction are observed between the instrumental variables and NTU indicators in the first-stage regression, and they pass the non-identifiability test. Additionally, the Wald F-statistic exceeds the critical value of 10%, rejecting the hypothesis of weak instrumental variables. This indicates that the selection of instrumental variables is in line with the requirements. In the results of the second-stage regression, the NTU indicator remains significantly positive, suggesting that the development of NTU can significantly promote the improvement of RPH. Compared with Table 2, the estimation results of 2SLS yield larger estimated coefficients, indicating that overlooking the endogeneity problem will underestimate the promotion effect of NTU on RPH.

**Table 5:** 2SLS regression results.

Variables	(1) First-stage regression	(2)
	NTU	RPH
*NTU*		0.688***	(15.13)
		*L.NTU*	0.918***	
(51.46)				
*Controls*	Yes	Yes
*Province FE*	Yes	Yes
*Year FE*	Yes	Yes
*Constant*	0.022	−0.039
	(1.17)	(−0.76)
Wald F statistic	926.60	
Observations	330	330
R-squared	0.974	0.735

***, **, and * represent significance levels of 1%, 5%, and 10%, respectively.

### 4.2 Threshold effects analysis

#### 4.2.1 Density of employment as a threshold variable

The number of thresholds for employment density is first identified, and a single-threshold effect is present. The single-threshold effects model is, in turn, regressed, and the results are provided (Table 6). The threshold value of employment density is 4.350. During the sample study period, the only region with an average employment density below 4.350 is Qinghai Province (4.342), while the rest of the regions are above the threshold value. Column (1) shows that as employment density crosses the threshold, the impact of NTU on RPH transforms from significantly negative to positive, with the regression coefficient changing from −0.196 to 0.463. During NTU, socio-economic agglomeration enhances employment, improves income through increased jobs, and enhances individual and public health. Verifying the findings of previous studies, NTU enhances the income of the floating population (43). With the increase in regional employment density, the promotion of RPH by NTU gradually increases, and H1a is supported.

**Table 6:** Threshold effect test results.

Variables	(1)	(2)
	Threshold variable: employment density	Threshold variable: advanced industrial structure
*NTU × I (llab ≤ 4.350)*	−0.196**	
	(−2.58)	
*NTU × I (llab > 4.350)*	0. 463***	
	(7.98)	
*NTU × I (struc ≤ 0.826)*		0.328***	(5.63)
		*NTU × I (0.826 < struc ≤ 1.744)*	0.414***	(7.37)
*NTU × I (struc > 1.744)*			0.581***	(10.05)
		Constant	−0.068	−0.163**
(−1.27)			(−2.22)
*Controls & fixed effects*		Yes	Yes
*R^2^*
0.806	0.761

***, **, and * represent significance levels of 1%, 5%, and 10%, respectively.

#### 4.2.2 Degree of advanced industrial structure as a threshold variable

First, the thresholds for the advanced industrial structure were identified, revealing a double-threshold effect. Second, the two-threshold effect model was regressed, and the results were obtained (column 2). Most of the area is in the range of 0.862 to 1.744. During the 2007–2018 period, the regions with an average industrial structure below 0.826 included the provinces of Anhui, Henan, Hebei, Jiangxi, Shaanxi, Qinghai, and Inner Mongolia, while the regions exceeding 1.744 are Beijing, Shanghai, and Hainan. The majority of regions fall between 0.862 and 1.744. The results in column (2) indicate that as the degree of advanced industrial structure crosses the threshold, the effect of NTU on RPH remains significantly positive, although the regression coefficient gradually increases. In other words, as the advanced industrial structure increases, the promotional effect of NTU on RPH exhibits a non-linear characteristic of gradual increase. It is further verified that H1b is proven. Upgrading the industrial structure is typically accompanied by efficiency gains and advancements in green technology. This can reduce resource consumption while enhancing economic and welfare outputs, decreasing polluting emissions, and optimizing the environment. Regional upgrades focus on minimizing negative impacts and maximizing desired outputs.

### 4.3 Heterogeneity analysis

#### 4.3.1 By geographic location

Considering China’s vastness, the resources and development patterns differ due to geographical variations. Consequently, there are differences in socio-economic conditions and development status as well. Based on the various economic regions, the sample is classified into east, central, and west, exploring the heterogeneity of NTU’s impact on public health. The results are shown in Table 7. NTU shows a positive impact on RPH across all regions, but the degree of impact is East < Central < West. In accordance with the heterogeneous results of NTU on reducing rural–urban disparities in existing studies (28), the magnitude of the effect varies across regions. China’s socio-economic development is likely to decrease from east to central and western regions. The eastern region, in general, is at the forefront of reform and has achieved advanced development, leading to limited benefits from NTU construction and a smaller contribution to public health enhancement compared to other regions. It has also been demonstrated that the influence of NTU on green growth is significantly heterogeneous, with significant differences in the suitability between NTU and resource allocation (22).

**Table 7:** Heterogeneity results.

Variables	(1)	(2)	(3)	(4)	(5)
	Eastern	Central	Western	Developing	Developed regions
*NTU*	0.111*	0.505***	0.643***	0.433***	0.069
	(1.69)	(5.58)	(4.00)	(5.22)	(1.10)
*Constant*	0.167	0.162*	0.250	0.248***	0.220**
	(1.58)	(1.94)	(1.51)	(2.91)	(2.33)
*Controls & fixed effects*	yes	yes	yes	yes	yes
*Obs*	144	144	72	204	156
*R^2^*	0.800	0.855	0.870	0.824	0.793

***, **, and * represent significance levels of 1%, 5%, and 10%, respectively.

#### 4.3.2 Classification by annual average GDP per capita

Considering the unbalanced regional socio-economic development in China, the 30 provinces are divided into 13 economically developed regions and 17 economically underdeveloped regions based on whether the annual average per capita GDP exceeds the national average. Due to their distinct development statuses and aggregated socio-economic resources, NTU’s impact on public health likely varies between economically developed and underdeveloped regions. From columns (4) and (5), NTU has a significantly positive impact on RPH in underdeveloped economic regions, which is consistent with the findings at the full-sample level, suggesting that NTU also promotes public health in these areas. However, in economically developed regions, NTU exhibits a positive but not statistically significant contribution to RPH. Two potential factors may explain this: first, in economically developed regions, where residents have higher income levels and human capital, it is challenging for NTU to influence RPH through the income effect. In contrast, in underdeveloped regions, per capita income is comparatively lower, allowing NTU to positively impact both individual and public health by increasing incomes. Second, public health services and medical resources are relatively well-established in areas with better economic development. The offsetting positive and negative effects of public health facility clustering make it difficult for NTU to significantly enhance RPH by constructing public health facilities.

### 4.4 Analysis of moderating effects

Economic and population agglomeration are represented in the aforementioned theoretical analysis as indicators of socio-economic conditions. The moderating effects model was used to examine the effects of economic and population agglomeration on NTU concerning public health (Table 8). Columns (2) and (4) present the results of adding cross-multiplier terms of economic and population agglomeration with NTU to the baseline regression, respectively. Column (2) shows that the interaction coefficient between economic agglomeration and NTU is significantly negative. NTU has a significant positive contribution to RPH enhancement, and the interaction coefficient (NTU × ecoagg) is negative after the interaction term is included.

**Table 8:** Results of moderating effects.

Variables	RPH			
	(1)	(2)	(3)	(4)
	Without interaction	With interaction	Without interaction	With interaction
*NTU*	0.272***	0.271***	0.325***	0.177***
	(5.48)	(5.48)	(8.08)	(2.83)
*Ecoagg*	0.060***	0.113***		
	(4.27)	(3.33)		
*NTU × ecoagg*		−0.130*		
		(−1.70)		
*Popu*			−0.090*	−0.285***
			(−1.90)	(−3.62)
*NTU × popu*				0.610***
				(3.08)
*Constant*	0.137**	0.117*	−0.017	0.060
	(2.19)	(1.86)	(−0.36)	(1.10)
*Controls & fixed effects*	Yes			
*R^2^*
0.803	0.804	0.778	0.784

***, **, and * represent significance levels of 1%, 5%, and 10%, respectively.

Meanwhile, the moderating variable (economic agglomeration) has a significantly positive coefficient. This indicates that the degree of economic agglomeration weakens the contribution of NTU to RPH, confirming Hypothesis 2a. Moreover, the positive role played by NTU is more pronounced with lower economic agglomeration, while the positive role of NTU gradually diminishes as economic agglomeration increases. In conclusion, economic agglomeration and NTU significantly substitute for each other in influencing RPH.

Columns (3)–(4) indicate that the main effect of NTU on RPH is significantly positive, and the moderating effect of population agglomeration with NTU is also positive, passing the significance test at the 1% level. As a moderating variable, population agglomeration significantly enhances this positive impact. Hypothesis 2b is, therefore, valid. The possible reasons are as follows: first, population agglomeration accelerates the quality of NTU and promotes socio-economic activities, providing favorable conditions for the improvement of RPH. Second, population agglomeration increases consumer demand and fosters the coordinated development of urban–rural linkages as well as the effective allocation of resources. It further promotes the popularization and upgrading of public services alongside improvements in residents’ health. Finally, reasonable population agglomeration supplies sufficient labor and encourages economic development, thereby enhancing residents’ income and living standards. In summary, population agglomeration and NTU have a synergistic effect on promoting the improvement of RPH.

### 4.5 Analysis of spillover effects

#### 4.5.1 Spatial autocorrelation

The spatial effect must be examined concerning spatial correlation, utilizing Moran’s *I* index (Table 9). With the exception of 2010, Moran’s *I* of NTU is significantly positive, indicating a strong spatial correlation among provinces during NTU promotion, with correlation values fluctuating above and below 0.300.

**Table 9:** Regional public health Moran’s index.

Year	Moran’s *I*	*p*-value	*Z*-value	Year	Moran’s *I*	*p*-value	*Z*-value
2007	0.164	0.087	1.709	2013	0.297	0.004	2.884
2008	0.200	0.041	2.041	2014	0.295	0.004	2.860
2009	0.366	0.001	0.366	2015	0.292	0.005	2.800
2010	0.094	0.250	1.151	2016	0.306	0.004	2.918
2011	0.284	0.005	2.792	2017	0.311	0.003	2.987
2012	0.300	0.003	2.951	2018	0.261	0.011	2.559

#### 4.5.2 Spillover effects

The SDM model was adopted to test the 0–1 spatial neighbor and economic distance weight matrix, with the results presented in Table 10. The spatial autocorrelation coefficients (rho) are significantly positive at both the 5 and 1% levels under both spatial weight matrices, indicating a significant positive spillover effect of RPH. This suggests that, on the one hand, with greater regional cooperation and integration, public health resources can be allocated more rationally within the region. Resources for medical services, education, and healthcare can be shared among different regions. On the other hand, the demonstration effect encourages neighboring regions to implement measures that enhance public health in the region, such as promoting healthy lifestyles and strengthening disease prevention.

**Table 10:** Results of spatial spillover effects.

Variables	(1)	(2)
	0–1 spatial adjacency matrix	Economic distance matrix
*NTU*	−0.138***	−0.089**	(−3.05)	(−2.00)
	W* NTU	−0.284***	−0.242**	(−2.80)	(−2.45)
*rho*		0.194**	0.203***	(2.57)	(2.68)
	*sigma2_e*	0.001***	0.001***	(13.36)	(13.35)
*Controls*		Yes	Yes
*City FE*	Yes	Yes
*Year FE*	Yes	Yes
*Observations*	360	360
*R-squared*	0.030	0.034
*Number of id*	30	30

***, **, and * represent significance levels of 1%, 5%, and 10%, respectively.

The negative coefficient of NTU and its spatial interaction term (W* NTU) on RPH indicates that the level of local public health is constrained by the NTU of neighboring regions. Possible reasons include, firstly, the cross-regional impact of ecological pollution. As urbanization accelerates, serious environmental pollution problems arise, with air and water pollution impacting public health in neighboring regions through mobility. Second, resources are unevenly distributed. During NTU, local areas may attract significant investment and resources, leading to a relative decrease in resources in adjacent areas. Consequently, this affects the investment of neighboring regions in public health sectors, including services and facilities, inhibiting improvements in public health levels. Third, there is unbalanced economic development. The economic growth resulting from the NTU may further widen the economic gap with neighboring regions. Meanwhile, economic backwardness often correlates with inadequate health care and insufficient access to health education, which can negatively influence public health levels.

To further analyze the influence and spillover characteristics of NTU on RPH, the effects should be decomposed into direct, indirect, and total effects (Table 11). In both spatial weighting matrices, all effects of NTU on RPH are negative. The indirect effects of NTU on RPH are stronger than the direct effects, indicating that NTU levels in neighboring regions have a greater inhibitory impact on RPH. Potential reasons include the fact that adjacent NTU development may lead to increased industrialization and the transmission of pollutants to the region through air movement, which raises health risks. Simultaneously, urbanization intensifies competition for public resources within neighboring areas, potentially attracting more public health resources, such as health and medical care, which creates resource constraints for locals and subsequently inhibits the improvement of public health services. Additionally, NTU promotes the sharing of resources and information within adjacent regions, and the lack of effective sharing may result in a squeezing effect on the health of neighboring areas. The estimated value of the adjacency matrix for the direct effect exceeds the economic distance, indicating that adjacency or lack thereof is a significant factor in the inhibitory role of NTU on regional public health.

**Table 11:** Decomposition of spatial effects.

Variables	0–1 spatial adjacency matrix			Economic distance matrix		
	Direct	Indirect	Total	Direct	Indirect	Total
*NTU*	−0.150*** (−3.09)	−0.371*** (−2.76)	−0.521*** (−3.23)	−0.099** (−2.09)	−0.313** (−2.37)	−0.413*** (−2.61)

***, **, and * represent significance levels of 1%, 5%, and 10%, respectively.

### 4.6 Limitations and future research directions

#### 4.6.1 Limitations

There are some limitations to this study. First, data access limitations exist. Although this study endeavored to collect data on multiple aspects of NTU, socio-economic agglomeration, and RPH, data on some key variables may not be comprehensive or could be missing due to the limited availability of data sources. Second, limitations in data processing may arise. The methodology used in data processing could have limitations that impact the results of the study. Third, there are limitations in research design. The study was based on a sample from a specific region or time period, which may not fully represent the broader situation, and the representativeness of the sample may limit the generalizability of the findings. Additionally, when constructing the research model, it may not be possible to cover all relevant variables, leading to omitted variable bias. Finally, the relationship between new-type urbanization and public health may be characterized by a time lag due to delays in infrastructure development, the delayed manifestation of environmental impacts, and intergenerational transmission of health behaviors resulting from new-type urbanization.

#### 4.6.2 Future research directions

To address the above possible limitations, future research directions can be developed in the following ways:

First, incorporate more up-to-date data. The NTU process and public health status have changed over time. Future research could incorporate more up-to-date data to reflect the impact of these changes on the relationship between NTU and public health. It will help to update and improve the findings of existing studies and the timeliness and accuracy of the research.

Second, other health dimensions are explored in depth. This study focused on the impact of NTU on public health, but public health is a complex and multidimensional concept. Future research can further explore the impact of NTU on other health dimensions (such as mental health and physical health) to fully reveal the comprehensive impact of NTU on public health. It helps to provide policymakers with a more comprehensive scientific basis to promote the coordinated development of urbanization and public health.

Third, by addressing issues such as omitted variable bias and measurement error, future research can improve research methods and models to increase the accuracy and reliability of the results. For example, more sophisticated statistical methods can be used to control the effects of omitted variables, and more precise measurement tools can be used to reduce measurement errors. Broaden data channels and explore and apply more advanced research methods, such as complex network analysis and spatial econometric models, to gain a deeper understanding of the complex relationship between NTU, socio-economic agglomeration, and RPH.

Fourth, exploring the heterogeneity among different regions by stratifying the analysis according to regional characteristics. The reliability of the research findings can be verified through robustness tests. Identify causal relationships using natural experiments or quasi-experimental designs to enhance the generalizability of research findings.

Finally, for possible time lag characteristics, the time lag structure can be incorporated into the analysis by selecting typical regions to carry out time lag effect tracking experiments and constructing open source time lag analysis toolkits.

## 5 Conclusion and policy implications

### 5.1 Conclusion

This study examines the effects and mechanisms of NTU on regional public health (RPH) by utilizing data from China between 2007 and 2018, constructing panel fixed-effects, threshold-effects, moderated-effects, and spatial spillover models. The study found that:

First, the direct effect shows that the “agglomeration effect” of NTU on RPH exceeds its “crowding effect” and has a significant driving effect overall. Employment density has a single threshold effect in the relationship between NTU and RPH. With employment density exceeding the threshold, the effect of NTU on RPH turns from negative to positive. The advanced industrial structure has a double threshold, and as the advanced industrial structure increases, the promotion effect of NTU on RPH presents a non-linear characteristic.

Second, the mechanism effect shows that socio-economic agglomeration plays an important moderating role in NTU and RPH. Specifically, there is a significant interaction between economic agglomeration and NTU in influencing RPH. Population agglomeration significantly increases NTU’s contribution to RPH, demonstrating a synergistic relationship with NTU.

Third, significant regional heterogeneity exists within NTU’s impact on RPH. Specifically, in the eastern, central, and western regions categorized by geographic location, NTU exerted a substantial positive influence on RPH, increasing in the order of influence. In economically developed and underdeveloped areas distinguished by socio-economic development, NTU’s impact on RPH is also positive. Notably, this effect is insignificant in economically developed regions.

Finally, spatial effect results reveal NTU’s significant negative spatial impact on regional PH, both under spatial proximity and economic distance weighting matrices. The decomposition effect indicates that under different spatial weight matrices, NTU’s direct, indirect, and total effects on RPH are all significantly negative, with the indirect effects being larger than the direct effects. By comparing the values of the estimated coefficients, adjacency emerges as the main factor in dampening the effect of NTU on RPH.

### 5.2 Policy implications

Based on the findings mentioned above, the following are the policy implications for the NTU strategy and its promotion of RPH development:

First, optimize the NTU strategy and strengthen the agglomeration effect. Future policies should focus on improving the quality of NTU, not just its speed and scale. Sustainability in urban development should be ensured through the optimization of urban planning and infrastructure development. Additionally, the policy should encourage industry diversification to create more employment opportunities while ensuring that employment density remains within rational limits to avoid the negative impacts of overcrowding. Further support should be directed toward high-technology industries, promoting the transformation and upgrading of traditional industries and enhancing added value and competitiveness.

Second, the moderating role of socio-economic agglomeration is considered. It is important to balance economic and population agglomeration, recognizing that economic agglomeration and NTU have a substitutive relationship in affecting RPH, while population agglomeration has a synergistic effect. Policies should rationally guide the spatial distribution of population and economic activities to achieve the coordinated development of economic and population agglomeration. Simultaneously, regional coordinated development should be strengthened to promote economic ties and cooperation among different regions, capitalizing on complementary regional advantages, narrowing regional development gaps, and improving overall public health.

Third, attending to regional heterogeneity and implementing differentiated policies is crucial. Eastern, central, and western regions, as well as economically developed and less developed areas, differ in the impact of NTU on RPH. Policies should develop differentiated NTU strategies and public health policies based on the characteristics and actual situations of different regions. In underdeveloped economic regions, NTU has a more significant role in promoting RPH. Policies should increase support for underdeveloped regions to advance their NTU process and enhance public health.

Fourth, regional cooperation should be strengthened to address spatial spillover effects. Future cross-regional cooperation mechanisms ought to be established to improve collaboration and exchanges between neighboring regions, working together to tackle the challenges posed by NTU. When formulating policies, it is essential to thoroughly consider the impact of spatial distance and economic ties on NTU and RPH.

Finally, the public health service system should be further strengthened. In NTU, attention should be paid to upgrading public health services, strengthening disease prevention and control, and raising residents’ health awareness and health literacy. Meanwhile, the social security system has been strengthened to ensure basic livelihood protection and medical care for residents during the NTU, reducing the health risks associated with urbanization.

## Data Availability

The original contributions presented in the study are included in the article/Supplementary material, further inquiries can be directed to the corresponding author.

## Footnotes

^1^The agglomeration effect is essentially a phenomenon of spatial concentration of economic activities, which manifests itself as the gathering of related industries or economic activities in a particular region, thus bringing about a series of economic effects and advantages (29). It can generate economies of scale, increase knowledge spillover, and pool labor, thus enhancing the overall competitive advantage of the regional economy.

^2^Threshold effect, is the phenomenon that when an economic variable reaches a certain threshold value, economic behavior or results will change significantly (31, 32). In macroeconomics, the threshold effect is mainly reflected in the study of economic growth and development.
