# Supplementary material for: New-type urbanization and regional public health: mechanisms and effects
Source: Front Public Health. 2025 Apr 24;13:1513173. doi: 10.3389/fpubh.2025.1513173 (PMC12058773; doi:10.3389/fpubh.2025.1513173)
Supplement: Supplementary file 1 [file Table_1.docx]

sum ph ur engry tech G H D L io

asdoc summarize ph ur engry tech G H D L io

gen lnpgdp =ln(pgdp)

*Panel space weight matrix construction

clear

cd D:

set matsize 360

use D:/weight_econ.dta

spcs2xt anhui- chongqing, matrix(W2) time(12)

spatwmat using W2xt,name(WW) standardize

*Debug Data Extractor

clear

use D:\202109-\data.dta

*Moran

spatwmat using D:/weight.dta,name(W2) standardize

preserve

spatgsa ur ,weights(W) moran twotail

**LM

reg ph ur engry engre_s tech G H D L io

asdoc reg ph ur engry engre_s tech G H D L io

spatdiag , weight(WW) //

asdoc spatdiag , weight(WW) //

est store m1

esttab m1 using LM检验.doc,r2 se star(* 0.1 ** 0.05 *** 0.01)replace nogap

***Hausman

spatwmat using W2xt,name(WW) standardize

xtset id year

xsmle ph ur engre_s tech G H D L ,fe wmat(W2) model(sdm) durbin(ur) nolog effects

est store sdm_fe //

xtset id year

xsmle ph ur engre_s tech G H D L, model(sdm) durbin(engry) wmat(W2) re nolog effects

est store sdm_re /

hausman sdm_fe sdm_re //

asdoc hausman sdm_fe sdm_re //

est store m1

esttab m1 using Hausman检验.doc,r2 se star(* 0.1 ** 0.05 *** 0.01)replace nogap

*Model robustness test - LR test

spatwmat using W2xt,name(WW) standardize

xsmle ph ur engre_s tech G H D L ,fe model(sdm) wmat(W2) type(time) nolog effects

est store sdm_a

xsmle ph ur engre_s tech G H D L ,fe model(sar) wmat(W2) type(time) nolog effects

est store sar_a

xsmle ph ur engre_s tech G H D L ,fe model(sem) emat(W2) type(time) nolog effects

est store sem_a

lrtest sdm_a sar_a //

lrtest sdm_a sem_a //

*Individual fixed effect VS time fixed effect VS double fixed effect

*Time fixed effect model

xtset id year

xsmle ph ur engry tech G H D L , fe model(sdm) wmat(W2) type(time) nolog noeffects

est store sdm_time

*Individual fixed effect model

xsmle ph ur engry tech G H D L,fe model(sdm) wmat(W2) type(ind) nolog noeffects

est store sdm_ind

*Double fixed effect model, spatial Durbin model

xtset id year

xsmle ph ur tech G H D , fe model(sdm) wmat(W2) type(both) nolog effects

est store sdm_both

lrtest sdm_both sdm_time,df(15) //

lrtest sdm_both sdm_ind, df(15) //

xtset id year

xsmle ph engry popu third io tech G H D L,wmat(W) model(sdm) type(both) dlag(3) nolog effects fe

**Spatial lag explains the direct, indirect and total effects of variables

xtset id year

gen lngreen1 = L.lngreen

xsmle ph engry popu third io tech G H D L, fe model(sdm) wmat(W) type(both) nolog effects robust

*Winsorization

help winsor2

winsor2 engry, cut(1 99) replace

*Intermediate effect test

gen struc = third/second

*Road infrastructure construction

xsmle ph engry popu io tech G H D , fe model(sdm) wmat(W) type(both) nolog effects

xsmle D engry popu io tech G H L , fe model(sdm) wmat(W) type(both) nolog effects

xsmle ph D popu io tech G H L, fe model(sdm) wmat(W) type(both) nolog effects

*so2

xsmle ph elec popu io tech G H D , fe model(sdm) wmat(W) type(both) nolog effects

xsmle lso2 engry popu io tech G H , fe model(sdm) wmat(W) type(both) nolog effects

xsmle ph lso2 popu io tech G H , fe model(sdm) wmat(W) type(both) nolog effects

*Adjustment effect test

xtset id year

xtreg ph ur ecod engry engre_s tech G H D L io , fe //

est store f1

gen J= ur* ecod //

xtreg ph ur ecod J engry engre_s tech G H D L io, fe

//The moderating effect of population agglomeration

xtset id year

xtreg ph ur popu engre_s tech G H D L io , fe //

est store f2

gen J2= ur* popu //

xtreg ph ur popu J2 engre_s tech G H D L io, fe

est store f2_jh

*Threshold effect test

xthreg ph tech H L D io , rx(ur) qx(struc) thnum(2) grid(100) trim( 0.01 0.01 ) bs( 300 300) r

drop if id ==2

drop if id ==3

drop if id ==5

drop if id ==12

drop if id ==13

drop if id ==15

drop if id ==17

drop if id ==18

drop if id ==21

drop if id ==24

drop if id ==26

drop if id ==29

drop if id ==30

//well-developed region

drop if id ==1

drop if id ==4

drop if id ==6

drop if id ==7

drop if id ==8

drop if id ==9

drop if id ==10

drop if id ==11

drop if id ==14

drop if id ==16

drop if id ==19

drop if id ==20

drop if id ==22

drop if id ==23

drop if id ==25

drop if id ==27

drop if id ==28

//Eastern Region

drop if id ==1

drop if id ==4

drop if id ==7

drop if id ==10

drop if id ==11

drop if id ==12

drop if id ==13

drop if id ==14

drop if id ==16

drop if id ==18

drop if id ==19

drop if id ==20

drop if id ==22

drop if id ==23

drop if id ==25

drop if id ==27

drop if id ==28

drop if id ==30

//Middle Region

drop if id ==2

drop if id ==3

drop if id ==4

drop if id ==5

drop if id ==6

drop if id ==7

drop if id ==8

drop if id ==9

drop if id ==15

drop if id ==17

drop if id ==19

drop if id ==20

drop if id ==21

drop if id ==24

drop if id ==26

drop if id ==27

drop if id ==28

drop if id ==29

//the west area

drop if id ==1

drop if id ==2

drop if id ==3

drop if id ==5

drop if id ==6

drop if id ==8

drop if id ==9

drop if id ==10

drop if id ==11

drop if id ==12

drop if id ==13

drop if id ==14

drop if id ==15

drop if id ==16

drop if id ==18

drop if id ==17

drop if id ==21

drop if id ==22

drop if id ==23

drop if id ==24

drop if id ==26

drop if id ==29

drop if id ==30
